# Machine learning-based prediction of response to Janus kinase inhibitors in patients with rheumatoid arthritis using clinical data

**DOI:** 10.3389/fimmu.2025.1689144

**Published:** 2025-11-26

**Authors:** Yeo-Jin Lee, Gyucheol Choi, Joongyeub Yeo, Jiyeong Baek, Heeju Choi, Minji Kim, Yong-Gil Kim, Bo Young Kim, Jamin Koo

**Affiliations:** 1 Department of Rheumatology, Asan Medical Center, University of Ulsan College of Medicine, Seoul, Republic of Korea; 2 ImpriMedKorea, Inc., Seoul, Republic of Korea; 3 Independent Researcher, Cresskill, NJ, United States; 4 ImpriMed, Inc., Mountain View, CA, United States; 5 Division of Rheumatology, Department of Internal Medicine, Gangneung Asan Hospital, University of Ulsan College of Medicine, Gangneung, Republic of Korea; 6 Department of Chemical Engineering, Hongik University, Seoul, Republic of Korea

**Keywords:** machine learning, precision medicine, Janus kinase inhibitor, rheumatoid arthritis, treatment response

## Abstract

**Objective:**

Rheumatoid arthritis (RA) is a chronic inflammatory disease with considerable heterogeneity in treatment response, leaving many patients unable to achieve remission or low disease activity. We aimed to develop a machine learning model to predict which patients with moderate-to-severe RA would respond to Janus kinase inhibitor therapy, thereby facilitating more effective and personalized treatment strategies.

**Methods:**

We retrospectively collected data from the Korean College of Rheumatology Biologics therapy (KOBIO) registry and Asan Medical Centers, including adult patients with moderate or high disease activity (DAS28-ESR≥3.2) and at least 12 months of follow-up. We trained and validated gradient boosting machine-learning models (XGBoost) to predict whether patients would achieve low disease activity or remission after 6 months of Janus kinase inhibitor therapy, using prespecified baseline covariates and stratified splits for independent training and test datasets.

**Results:**

This study included 264 patients with moderate-to-severe rheumatoid arthritis from the Korean cohorts (the KOBIO registry and Asan Medical Centers). Of these, 247 received either tofacitinib (n=123) or baricitinib (n=124). After 6 months of treatment, 65% of patients on tofacitinib and 70% on baricitinib achieved low disease activity or remission. Our machine-learning models (trained and validated separately for each drug) achieved high predictive performance (tofacitinib: ROC-AUC 0.82, accuracy 80%; baricitinib: ROC-AUC 0·88, accuracy 88%), identifying key clinical factors such as total cholesterol, CRP, and specific joint swelling or tenderness for tofacitinib, and patient global assessment, joint swelling, and co-administration of hydroxychloroquine for baricitinib. Model-guided treatment selection could have improved outcomes for an additional 15% of patients by aligning each individual’s predicted response with the more suitable Janus kinase inhibitor.

**Conclusion:**

The findings suggest that ML models can accurately predict treatment response to Janus kinase inhibitors in rheumatoid arthritis and may support personalized therapy selection to improve clinical outcomes.

## 1 Introduction

Rheumatoid arthritis (RA) is a chronic inflammatory disease affecting multiple joints, leading to disability and deformity (1). Treat-to-target strategies for RA aim to achieve remission or low disease activity through frequent monitoring and individualized medication adjustments (2). Despite improved clinical outcomes for RA, a considerable proportion of patients experience treatment failure. Studies indicate that 60% of patients with RA do not achieve treatment goals with conventional synthetic disease-modifying anti-rheumatic drugs (csDMARDs), while 30–40% fail to respond to biological DMARDs (bDMARDs) (3–6). Treatment failure imposes significant burdens, including high drug costs, adverse effects, and poor clinical outcomes (7–9). Given the variability in treatment response and challenges in selecting DMARDs with unequal mechanisms of actions (MoAs), predicting which patients will respond to a given therapy could facilitate rapid achievement of therapeutic targets and personalized DMARD selection.

With the growing availability of structured clinical data and advances in computation methods, machine learning (ML) has emerged as a promising approach in clinical informatics to support individualized treatment selection. Prior ML studies in RA have focused on a disease activity forecasting or treatment response prediction, primarily for csDMARDs and bDMARDs (10, 11). A small number of researchers have investigated tumor necrosis factor inhibitor response using genetic markers, but these approaches tend to be costly and challenging to implement broadly (12, 13). Recent ML-based efforts utilizing clinical data also remain mostly confined to bDMARDs (14–16). To our knowledge, no ML models aimed at predicting RA patients’ response to an Janus kinase inhibitor (JAKi) have been reported thus far.

JAKis have demonstrated efficacy in patients with inadequate responses to csDMARDs or bDMARDs, showing superiority to methotrexate and non-inferiority or superiority to adalimumab or abatacept (17). Additional benefits include inhibition of radiographic progression and improvements in patient-reported outcomes (18–20). Given their strong clinical preference for RA treatment, predicting JAKi response prior to administration is crucial for reducing treatment failures and improving patient outcomes. In this study, we aimed to develop and validate ML models that predict treatment response to individual JAK inhibitors, specifically tofacitinib and baricitinib, using routine clinical data. We also sought to identify key baseline features associated with response and to assess the potential clinical utility of model-guided treatment stratification.

## 2 Methods

### 2.1 Patient cohort and datasets

**Figure 1:**
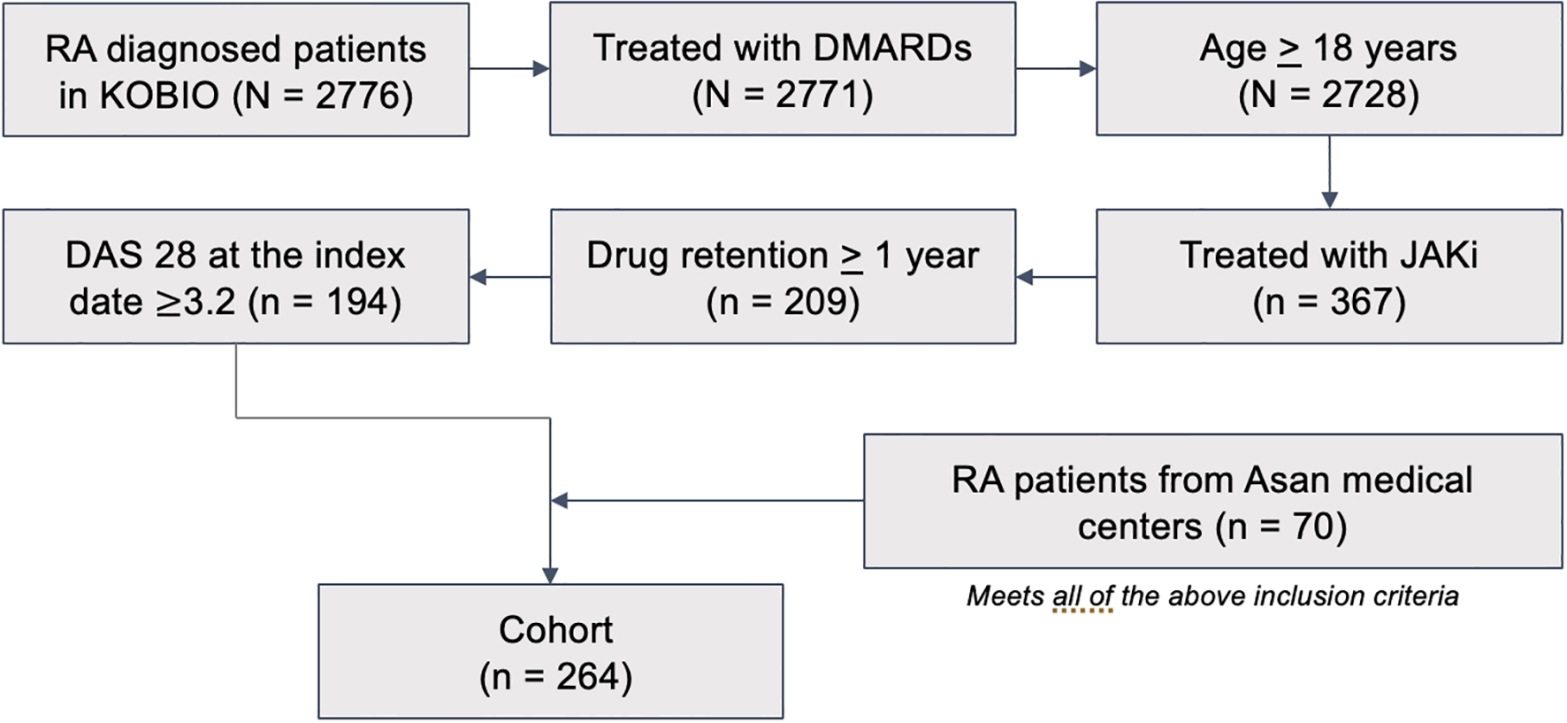
Patient selection flow chart.

The initial dataset was retrieved from the registry of Korean College of Rheumatology Biologics therapy (KOBIO). A total of 194 patients met all of the following inclusion criteria: 1) at least 18 years old; 2) treated with DMARD(s) prior to receiving any of the bDMARD(s); 3) treated with JAKi and at least 12 months of follow-ups available; 4) initially at the status of moderate or high disease activity (**Figure 1**). Patients with other systemic autoimmune rheumatic diseases (e.g., lupus, psoriatic arthritis, ankylosing spondylitis) were also excluded from the study cohort. Records of 229 covariates were available in the registry of KOBIO. The additional dataset of 70 RA patients who met all of the aforementioned inclusion criteria was retrieved from the Asan Medical Centers (AMC, Republic of Korea). This study was approved by the Institutional Review Board (IRB) of the Gangneung Asan Hospital (IRB #GNAH-2023-02-009) and AMC (IRB #2024-1048). Informed consent was waived due to the retrospective analysis of the anonymized datasets.

### 2.2 Analysis of treatment response

Treatment responses were evaluated by comparing Disease Activity Score in 28 joints using the erythrocyte sedimentation rate (DAS28-ESR) of the patient at the index date (or closest date prior to administration of drug(s)) and at the date 6 months after the index date (or the closest date not surpassing 12 months). DAS28-ESR scores were converted into the categorical labels on the disease status—remission, low disease activity, moderate disease activity, or high disease activity—according to the international standards (21). Positive response to the drug (JAKi) was defined as the change in the status from moderate or high disease activity to low disease activity or remission (DAS28-ESR <3.2). All other changes in the status were classified as lack of response including disease progression.

### 2.3 Development of machine learning models

102 covariates were readily available in both the KOBIO and AMC registry. These covariates consisted of diverse kinds, ranging from demographics including age and sex to comorbidities such as hypertension and heart arrhythmia. In terms of the data type, 66 and 36 variables were categorical or numerical, respectively. Categorical variables were converted into numerical variables via label encoding (*scikit-learn* v.1.2.2) (22). The numerical variables were re-scaled using the robust scaler (*RobustScaler*). Missing data were left as is, meaning the patients with missing values in some of the covariates were still included in the dataset used to train or test ML models.

We first evaluated effectiveness of the various ML methods via AutoML and then developed the models using XGBoost (eXtreme Gradient Boosting v.1.7.1) (23, 24). XGBoost is one of the most popular, non-linear, and flexible methods that handles missing data and categorical features effectively, without the need for imputation or manual encoding. The models were trained to predict whether a patient will achieve a positive response after 6 months when treated by a given JAKi. ML models utilizing the minimal subset of variables were selected from the ones achieving the highest ROC-AUC during 5-fold cross validation (25). To further prevent overfitting, an early stopping rule was applied where we stopped the training process when the improvement of Receiver Operating Characteristics Area Under the Curve (ROC-AUC) is less than 0.01 in the validation set. We computed Shapley Additive exPlanations (SHAP) values using the XGBoost TreeExplainer. SHAP values were calculated on held-out test sets from each cross-validation fold and visualized with summary plots and patient-level force plots.

Predictive performance of the ML models was evaluated using the independent, *unseen* dataset that had been split prior to the training process. In order to guarantee similarity between the training and test datasets, the datasets from the KOBIO and AMC registry were merged and then split into the training and test datasets by the method (*train_test_split*) that preserves the ratio of labels (treatment response) (22). For the chosen ML models, the thresholds for classifying a treatment response were chosen to maximize F1 score (26). The test performance was assessed with respect to accuracy, positive predictive value (PPV), and negative predictive value (NPV).

### 2.4 Statistical analysis

Clinical and molecular characteristics of the patient subgroups were compared using the Fisher’s exact test or chi-square test for categorical variables and a two-sample t-test or Mann–Whitney U test for continuous variables, respectively. *P* values were corrected using Bonferroni method when multiple tests were indicated. Multi-variable logistic regression was performed with respect to exhibiting a positive response to tofacitinib or baricitinib. The significance level was set at *P* < 0.05. Analyses were performed using Python (version 3.9.7) or Prism 8 (GraphPad, San Diego, CA, USA).

## 3 Results

### 3.1 Patient selection

This study included 264 RA patients who received JAKi therapy between 2018 and 2023 (**Figure 1**). The median age was 59 years and 78% of the cohort were female. When comparing the patient characteristics of the original datasets from two registries, we observed significant differences in several baseline characteristics including age and smoking history (**Table 1**). Consequently, we combined the datasets into a single cohort and performed a stratified split into training and test datasets to enhance robustness of the ML model performance. Before splitting, the RA patients were grouped by treatment—tofacitinib (n=123), baricitinib (n=124), or upadacitinib (n=17). We focused on the first two groups (N = 247) for model development as the upadacitinib group was too small for translational analysis. Notably, the positive response rates of these two groups were similar—65% for tofacitinib versus 70% for baricitinib (**Supplementary Figure 1**). The characteristics of the patients were also insignificantly different between the training and test datasets in each treatment group (**Supplementary Tables 1**, **2**), including the background treatment status represented by the proportions of patients receiving csDMARDs prior to JAK inhibitor initiation.

**Table 1:** Baseline demographic and clinical characteristics of the study cohort.

Characteristic	KOBIO registry (n = 194)	AMC registry (n = 70)	*P* value
Age at index date, yrs median, range	57, 22–86	61, 37–82	0.05
Sex			1.0
Female	160 (82%)	56 (80%)	
BMI, kg/m^2^			0.32
≤30	184 (95%)	69 (99%)	
>30	10 (5%)	1 (1%)	
Smoking history			0.04
Ex-smoker	10 (5%)	10 (14%)	
Current smoker	20 (10%)	5 (7%)	
Never smoked	164 (85%)	55 (79%)	
Comorbidities			
Hypertension	53 (27%)	17 (24%)	0.7
Ischemic heart disease	4 (2%)	3 (4%)	0.6
Hyperlipidemia	41 (21%)	23 (33%)	0.07
Diabetes	23 (12%)	4 (6%)	0.3
Renal failure	2 (1%)	0 (0%)	0.96
csDMARDS at index date			
any csDMARD	185 (95%)	60 (86%)	0.01
MTX only	159 (82%)	51 (73%)	0.12
csDMARDs excluding MTX	100 (52%)	39 (56%)	0.58
DAS 28 at index date			
median, range	5.4, 3.3–7.8	5.4, 4.0–7.1	0.3

KOBIO, Korean College of Rheumatology Biologics therapy; AMC, Asan Medican Center; BMI, body mass index; DAS, disease activity score; DMARD, disease-modifying antirheumatic drug; MTX, methotrexate.

### 3.2 Response analysis

**Figure 2:**
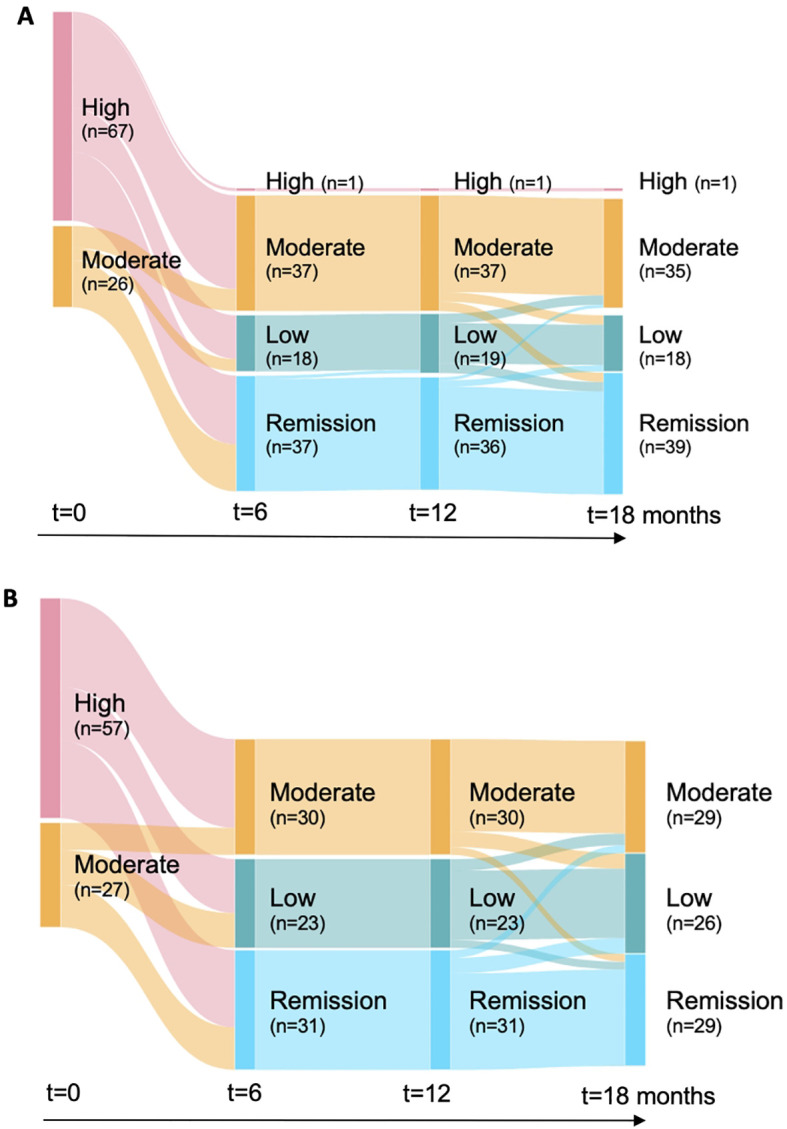
Dynamic changes in the disease activity status of the RA patients. Observations among the patients treated by **(A)** tofacitinib or **(B)** baricitinib. The analysis was done with respect to the patients of only the KOBIO registry as the time-course of assessment results were not available from the AMC registry.

DAS 28-ESR scores following treatment initiation were available across multiple time points with uneven intervals (**Supplementary Figure 2**) in the KOBIO registry, which captures real-world data from approximately 2, 800 RA patients treated at 47 academic and community rheumatology centers across S. Korea. As shown in **Figure 2**, the greatest change in disease status—based on DAS 28-ESR scores—occurred within the first six months of treatment. Disease status distributions were similar between the two treatment groups at the index date, with the majority (66%) presenting high disease activity. Within the first six months of JAKi therapy, 64% of the patients achieved low disease status or remission. Overall, 96% of the patients who ever reached low disease status or remission did so within those initial six months, whereas only 4% did so by 18 months, and 3% progressed to moderate disease status. Based on these observations, we developed ML models to predict the probability of positive response, i.e., low disease status or remission, within the first 6 months.

### 3.3 ML-based response prediction and stratification

**Figure 3:**
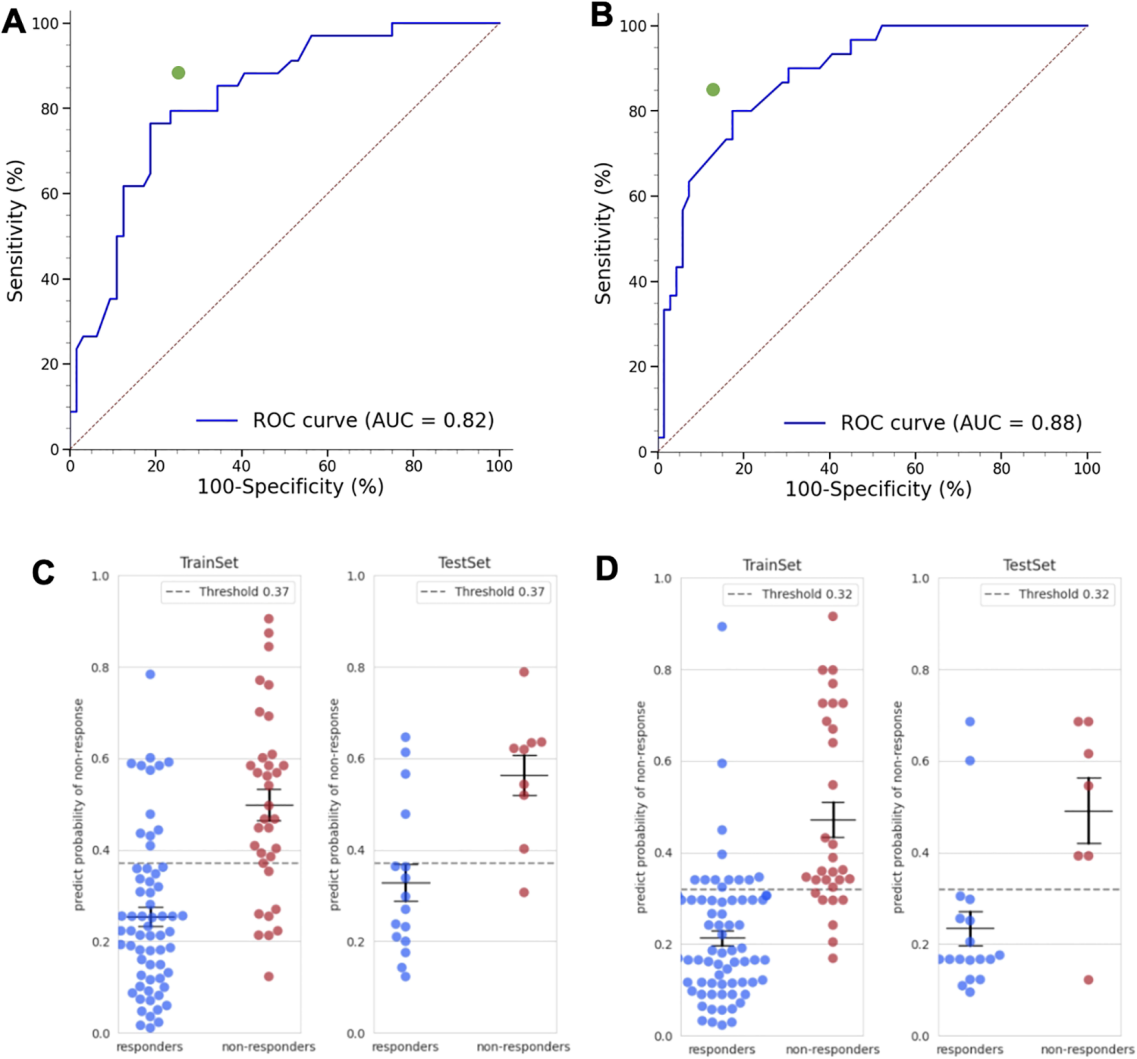
Predictive performance of the ML models. ROC-AUC and test accuracies of the ML models predicting positive response to **(A)** tofacitinib or **(B)** baricitinib. The distribution of the probability of non-response estimated by the **(C)** tofacitinib or **(B)** baricitinib model. The left and right figures represent the results for the training and test dataset, respectively.

We developed two ML models to predict the likelihood of a positive response to tofacitinib or baricitinib. Using the training dataset for the tofacitinib group, the model achieved a validation ROC-AUC of 0.82. Six covariates—total cholesterol (normal vs abnormal), C-reactive protein level, DAS 28-ESR score (ERP) at the index date, swelling at left knee joint, right 4^th^ PIP joint, and tenderness of right 2^nd^ MTP joint (TRMT2)—were used by the final model, yielding a test accuracy of 80% (**Figure 3**). The importance of these covariates in predicting a positive response to tofacitinib varied, with total cholesterol being the most important, followed by CRP (**Supplementary Figure 3**). We repeated stratified 5-fold cross-validation with shuffling across 10 runs and observed a mean ROC-AUC of 0.79 with standard deviation (SD) of 0.06, indicating consistent performance across data splits (**Supplementary Figure 4**). We also performed SHAP analysis on representative patients. These patient-level visualizations demonstrated how individual feature contributions shaped the predicted probability of treatment response, offering intuitive, case-specific insights beyond aggregate feature importance rankings (**Supplementary Figure 5**). Calibration analysis indicated a good probability accuracy (Brier score 0.16) with reliability shown in **Supplementary Figure 6**. Decision-curve analysis indicated higher net benefit than treat-all or treat-none strategies across clinically relevant thresholds (**Supplementary Figure 7**).

Using the ML-estimated likelihood of a positive response to tofacitinib (**Figure 3C**), we classified the entire cohort (including those treated with baricitinib) into improbable responders with a low likelihood of response (tofacitinib^NR^; no response, NR) and probable responders with a high likelihood of response (tofacitinib^PR^; positive response, PR). The baseline characteristics of these two subgroups were largely similar except for sex, where a significantly higher proportion of female was noted in the tofacitinib^NR^ subgroup (**Table 2**). In contrast, covariates used by the ML model showed notable differences between tofacitinib^NR^ and tofacitinib^PR^ except for total cholresterol, with the largest discrepancy observed for CRP. Multivariate analysis (**Supplementary Figure 8**) also showed that baseline characteristics had only a limited—and largely insignificant—influence on the likelihood of a positive response to tofacitinib.

**Table 2:** Comparison of the patients predicted as tofacitinib^PR^ or tofacitinib^NR^.

Characteristic	tofacitinib^PR^ (n = 73)	tofacitinib^NR^ (n = 50)	*P* value
Age at index date, years			0.22
median, range	59, 35–77	55, 34–77	
Sex			0.05
Male	19 (26%)	5 (10%)	
Female	54 (74%)	45 (90%)	
BMI, kg/m^2^			1.00
≤30	69 (95%)	48(96%)	
>30	4 (5%)	2 (4%)	
Smoking history			0.11
Ex-smoker	6 (8%)	2 (4%)	
Current smoker	10 (14%)	2 (4%)	
Never smoked	57 (78%)	46 (92%)	
Comorbidities			
Hypertension	21 (29%)	11 (22%)	0.53
Ischemic heart disease	1 (1%)	1 (2%)	1.00
Hyperlipidemia	15 (21%)	11 (22%)	1.00
Diabetes	11 (15%)	5 (10%)	0.59
Renal failure	1 (1%)	1 (2%)	1.00
DAS 28 at index date			
median, range	5.4, 3.6–7.1	5.6, 3.4–7.2	0.29
CRP^a^, mg/dL median, range	2.11, 0.1-27.6	0.36, 0.03-69.7	<0.001
Total cholesterol^a^			
Normal	52 (80%)	23 (82%)	1.00
Abnormal	13 (20%)	5 (18%)	
Number of swelling joints^a^ median, range	5.0, 0.0-14.0	6.5, 1.0-30.0	0.12
Number of tendern joints^a^ median, range	6.0, 0.0-22.0	n7.0, 1.0-29.0	0.41

a The features listed in the table include those used by the ML model in predicting the likelihood of a positive response to tofacitinib.

BMI, body mass index; DAS, disease activity score; CRP, C-reactive protein.

The ML model trained on the baricitinib treatment dataset yielded a validation ROC-AUC of 0.88, with a test accuracy of 88% (**Figure 3B**). An identical robustness analysis yielded a mean ROC-AUC of 0.80 (SD = 0.08) across 10 runs for the baricitinib model, further supporting stability to resampling (**Supplementary Figure 5**). Calibration analysis likewise showed good probability accuracy (Brier score 0.14) with reliability shown in **Supplementary Figure 6**. Again, decision-curve analysis indicated higher net benefit than treat-all or treat-none strategies across clinically relevant thresholds (**Supplementary Figure 7**). Seven covariates—largely distinct from those predicting response to tofacitinib—were used: The patient’s global assessment of the disease status (PtGA), swelling at the right IP joint (SRIP), left elbow joint (SLEL), left wrist joint, left, right 1st MCP joint (SRMC1), or right 5^th^ MCP joint (SRMC5), tenderness at left AC joint (TLAC), and co-administration of hydroxychloroquine. The most important covariates were SLEL and SRIP, followed by SRMC1 and SRMC5 (**Supplementary Figure 3**). Using the final model’s estimated likelihood of a positive response (**Figure 3D**), we again stratified the cohort into improbable (baricitinib^NR^) and probable (baricitinib^PR^) responders. Baseline characteristics between these two subgroups were largely similar (**Table 3**), and we also observed insignificant differences among the covariates used by the ML model except for the patient’s global assessment. The multivariate analysis indicated that baseline characteristics had only a limited impact on the likelihood of a positive response to baricitinib (**Supplementary Figure 8**).

**Table 3:** Comparison of the patients predicted as baricitinib^PR^ or baricitinib^NR^.

Characteristic	baricitinib^PR^ (n = 80)	baricitinib^NR^ (n = 44)	*P* value
Age at index date, years			0.16
median, range	60, 22–82	56, 25–74	
Sex			0.42
Male	15 (19%)	5 (11%)	
Female	65 (81%)	39 (89%)	
BMI, kg/m^2^			0.79
≤30	76 (95%)	43(98%)	
>30	4 (5%)	1 (2%)	
Smoking history			0.15
Ex-smoker	10 (12%)	1 (2%)	
Current smoker	7 (9%)	5 (11%)	
Never smoked	63 (79%)	38 (86%)	
Comorbidities			
Hypertension	20 (25%)	12 (27%)	0.83
Ischemic heart disease	3 (4%)	1 (2%)	1.00
Hyperlipidemia	26 (32%)	8 (18%)	0.10
Diabetes	8 (11%)	2 (5%)	0.32
Renal failure	0 (0%)	0 (0%)	
DAS 28 at index date			
median, range	5.3, 3.5–7.8	5.4, 3.5–6.9	0.43
Patient’s global assessment median, range	8.0, 2.0-10.0	7.0, 4.0-9.0	<0.001
Hydroxychloroquine^a^			0.93
Co-administration	2 (2%)	2 (5%)	
None	78 (98%)	42 (95%)	
Number of swelling joints^a^ median, range	5.0, 0.0-19.0	5.0, 0.0-16.0	0.80
Number of tendern joints^a^ median, range	7.0, 0.0-31.0	6.0, 0.0-21.0	0.94

a The features listed in the table include those used by the ML model in predicting the likelihood of a positive response to tofacitinib.

BMI, body mass index; DAS, disease activity score.

### 3.4 Potential improvement in treatment outcomes

**Figure 4:**
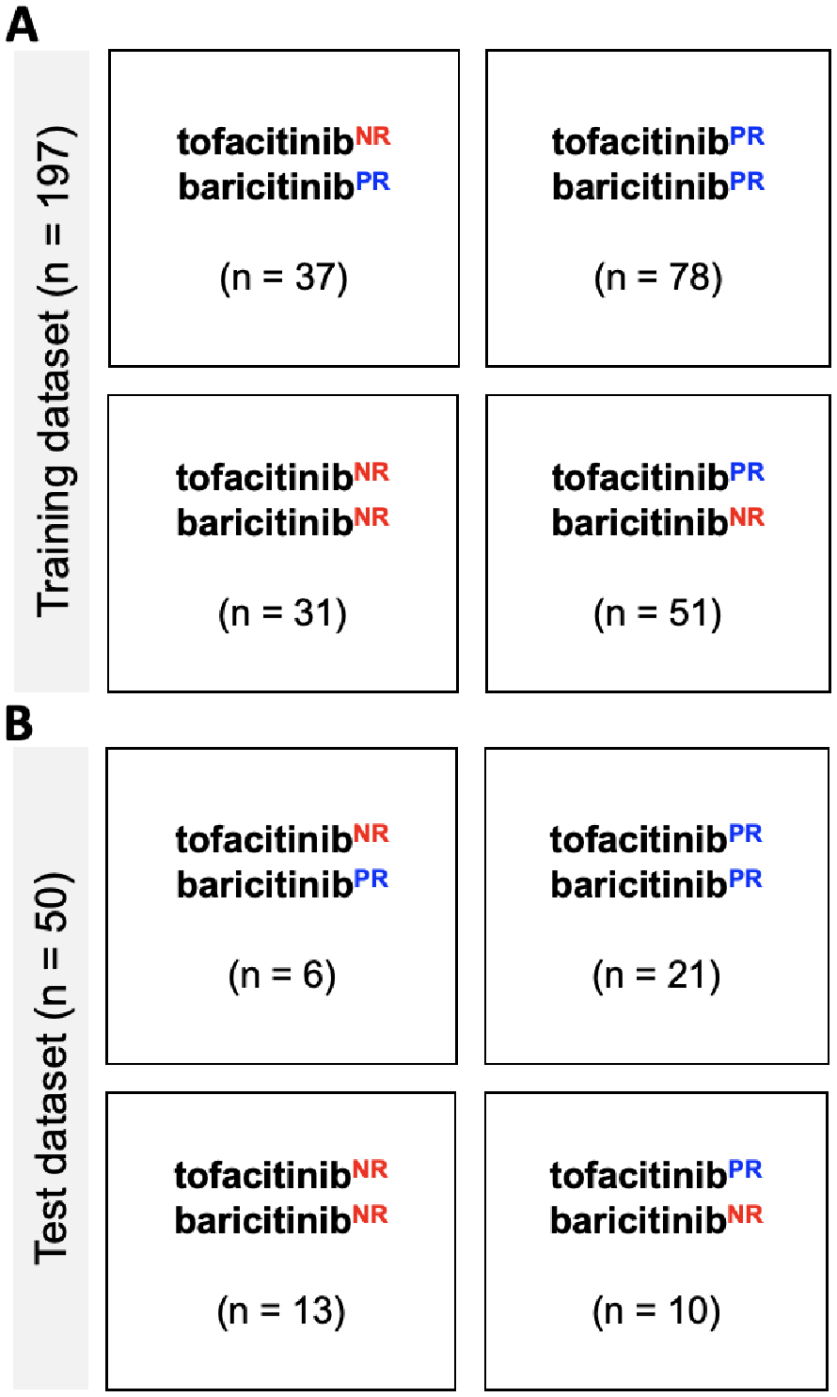
Combinatorial classification of the RA patients based on the predicted response to the two JAKi drugs. Classification results of the patients in the **(A)** training and **(B)** test dataset.

We applied the trained models to understand how the ML models may help to improve clinical outcomes of the entire cohort. For this purpose, we classified the RA patients into four subgroups in terms of the predicted response to the two JAKi drugs (**Figure 4**). When applying the trained model to the baricitinib treatment group, 87 (65%) of the patients were predicted as tofacitinib^PR^. Among these patients, 25 (17%) were predicted as baricitinib^NR^ and did not respond positively to baricitinib. Similarly, application of the ML model to the tofacitinib treatment group revealed that 62 (50%) of the patients were baricitinib^PR^, among whom 15 (12%) were tofacitinib^NR^ and did not benefit from tofacitinib. These analyses suggest that the application of the proposed methodology may have contributed to improved treatment outcome of 40 (15%) of the cohort by assisting selection between the two JAKi drugs.

## 4 Discussion

Our ML models demonstrated high predictive performance, achieving an AUC-ROC of 0.82 for tofacitinib and 0.88 for baricitinib. On an independent test dataset, model accuracies were 80% and 88%, respectively. Compared with previous studies predicting bDMARD response using XGBoost, our models maintained high accuracy and AUC despite differences in outcome definitions (remission based on DAS 28 vs ACR 20) (14, 15). Moreover, these results are comparable to those of a recent study that predicted low disease activity or remission in RA patients treated with bDMARDs (accuracy 0.851, AUC-ROC 0.910) (16). In our cohort, the overall response rates for tofacitinib and baricitinib were 65% and 70%, respectively. Given the high, robust predictive accuracy, ML-based prediction could enhance clinical utility by guiding treatment decisions for the two-thirds of patients likely to respond, while reducing failures in the remaining one-third. Although this study did not assess cost implications, previous research revealed that precision medicine approaches for RA can reduce healthcare expenses by anticipating responses and avoiding ineffective treatments (27).

JAKis are targeted synthetic DMARDs that inhibit intracellular cytokine signaling. Differences in signal transducer and activator of transcription (JAK-STAT) combinations can affect cytokine pathways and immune modulation, potentially leading to distinct therapeutic effects among JAKi agents (17). Tofacitinib primarily inhibits JAK1 and JAK3, whereas baricitinib has a stronger affinity for JAK1 and JAK2. In our study, the two agents resulted in unequal overall response rates (65% vs 70%), predictive performance (AUC-ROC of 0.82 vs 0.88), and clinical factors influencing treatment response. These findings suggest that ML-based prediction of JAKi response in RA may help clinicians select the most suitable agent. Moreover, our results indicate that predicting response before JAKi initiation can improve treatment outcomes in an additional 15% of patients by guiding selection between the two JAKi agents.

Our ML models identified key clinical features associated with achieving remission or low disease activity following JAKi therapy. The most influential variables were predominantly related to RA disease activity. In the tofacitinib group, all significant variables except total cholesterol were directly associated with disease activity, i.e., CRP, tender joint, swollen joint, and DAS 28-ESR. Although CRP is not formally part of the DAS 28-ESR calculation, it remains a widely used marker of inflammation and is included in DAS 28-CRP (28). Total cholesterol is also an indirect indicator of RA disease activity: Inflammatory states involving cytokines, and the subfraction and function of blood lipids (including total cholesterol) are altered and contribute to lipid metabolism abnormalities in RA (29). In the baricitinib group, PtGA emerged as an important variable for the ML model alongside tender and swollen joint. PtGA is a core component of the DAS 28 score and has previously been highlighted as a key clinical factor in predicting bDMARD response (15). Other patient-reported measures such as patient index data 3 (RAPID3) have also proven valuable in forecasting bDMARD outcomes in RA (30). In this regard, these findings underscore the significance of both clinical markers of disease activity and patient-reported outcomes in modeling JAKi treatment responses.

Beyond these systemic factors, several joint-specific variables (e.g., tenderness or swelling of individual joints) were identified as important predictors in our ML model. These features should be interpreted with caution, as they may represent data-driven correlates rather than biologically validated markers. Due to the inherent characteristics of ML-based prediction models, such localized features were automatically selected based on their contribution to predictive performance rather than predefined biological hypotheses. Nonetheless, recent studies have highlighted that anatomical variations in RA pathophysiology may contribute to differential treatment responses across affected joints (31). Consistent with this, hand-dominant involvement has been associated with a more favorable prognosis, whereas foot involvement is linked to poorer outcomes, likely reflecting histological differences among joint types (32). Therefore, while the predictive contribution of individual joint features may reflect localized disease activity patterns, further biological validation is warranted to determine their mechanistic relevance.

The observed association between lipid parameters or CRP and JAKi treatment response may be partly explained by the biological functions of the JAK–STAT pathway in lipid metabolism and inflammation. This pathway mediates signaling for more than 50 cytokines and growth factors that regulate immune and metabolic homeostasis (33). Pharmacologic inhibition of this pathway has consistently been associated with elevations in serum lipid parameters across both clinical trials and experimental models (34–36). Mechanistically, activation of type I and II interferon–dependent JAK/STAT signaling in macrophages downregulates cholesterol biosynthesis and uptake while promoting cholesterol esterification, contributing to the hypolipidemic phenotype characteristic of systemic inflammation (37). Inhibition of JAK1 signaling by tofacitinib or other JAK inhibitors may reverse these effects, restoring lipid homeostasis and resulting in increased circulating cholesterol levels. Furthermore, JAK/STAT signaling is integral to adipocyte differentiation and lipid metabolism, as STAT1, STAT3, STAT5A, and STAT5B are expressed in adipocytes and modulate adipogenesis and lipid storage (38). Additional evidence suggests that attenuation of IL-6–mediated signaling reduces inflammation-induced lipid catabolism while enhancing lipid efflux through upregulation of liver X receptor α (LXRα) and ATP-binding cassette transporter A1 (36). Moreover, CRP synthesis in hepatocytes is transcriptionally regulated via the IL-6–JAK/STAT axis (39, 40), providing mechanistic plausibility for the inclusion of cholesterol and CRP as a predictive variable in our ML model.

Recent advances in ML and insufficient treatment response of RA have prompted numerous studies predicting treatment response in RA (41, 42). However, most focused on csDMARDs or bDMARDs, and ML-based prediction for targeted synthetic DMARDs (tsDMARDs), such as JAK inhibitors, remains limited. A recent ML study predicted difficult-to-treat RA without differentiating between bDMARDs and tsDMARDs (43), while another used conventional logistic regression to analyze factor associated with JAK inhibitor response (44), lacking ROC-AUC or predictive accuracy metrics and clinical applicability. In contrast, our study is among the first to develop and validate ML models predicting clinical response to tofacitinib and baricitinib, demonstrating the feasibility of precision medicine for tsDMARDs in RA. Moreover, the proposed approach and ML models achieved consistently high accuracy in both the development and test datasets for predicting response to JAKi therapy. Previous studies have shown that tofacitinib and baricitinib are effective in patients who exhibit incomplete responses to csDMARDs or bDMARDs (18, 45–49). JAKi therapy is also considered a favorable option for difficult-to-treat RA cases, where multiple biological and targeted synthetic DMARDs have failed (50). Accordingly, our ML models address an unmet need associated with administering these potent agents to patients with severe RA.

Several limitations need to be considered before integration into clinical practice. First, although our ML models demonstrated high accuracy and AUC-ROC values, their performance must be validated in larger, ethnically and geographically diverse cohorts. Because our study population was limited to Korean patients, external validation using independent, international datasets will be essential to confirm generalizability. Second, while our models focused on clinical variables, they did not explore biological mechanisms underlying differential drug responses. Integrating molecular and cellular data in future studies may help elucidate the immunological basis of treatment response. Third, our inclusion criteria may have encompassed patients with refractory RA who had failed multiple DMARDs, including at least one bDMARD (51, 52), which should be considered when interpreting the predictive outcomes and related clinical factors. Finally, missing values in some covariates may have influenced both the predictive algorithm and the analysis results.

## 5 Conclusion

We developed ML models capable of accurately predicting JAK inhibitor treatment response in patients with active RA, while also pinpointing key clinical variables that influence therapeutic outcomes. These findings extend our understanding of the clinical determinants underlying JAKi efficacy and may facilitate more personalized treatment strategies aimed at minimizing ineffective therapies. Looking ahead, future work will focus on validating these models across international cohorts and extending them into a multi-modal framework that integrates clinical, transcriptomic, proteomic, and immunophenotypic data. Such integration will not only enhance predictive accuracy but also help uncover the biological mechanisms underlying JAK inhibitor response, ultimately bridging data-driven prediction with mechanistic understanding in clinical immunology.

## Data Availability

The test dataset will be made available by the authors upon request.
