# Supplementary material for: Machine learning-based prediction of response to Janus kinase inhibitors in patients with rheumatoid arthritis using clinical data
Source: Front Immunol. 2025 Nov 26;16:1689144. doi: 10.3389/fimmu.2025.1689144 (PMC12689586; doi:10.3389/fimmu.2025.1689144)
Supplement: Supplementary file 1 [file Table1.docx]

**Supporting Information for**

Machine learning-based prediction of response to Janus kinase inhibitors in patients with rheumatoid arthritis using clinical data

Yeojin Lee^1,+^, Gyucheol Choi^2,+^, Joongyeub Yeo^3^, Jiyeong Baek^2^, Heeju Choi^4^, Minji Kim^2^, Yong-Gil Kim^1^, Bo Young Kim^5,*^, Jamin Koo^2,4,6,*^

^1^Department of Rheumatology, Asan Medical Center, University of Ulsan College of Medicine, 88 Olympic-ro 43-gil, Songpa-gu, Seoul, 05505, Republic of Korea

^2^ImpriMedKorea, Inc., Seoul 03920, Republic of Korea

^3^Independent Researcher, NJ 07650, United States of America

^4^ImpriMed, Inc., Mountain View, CA 94043, United States of America

^5^Division of Rheumatology, Department of Internal Medicine, Gangneung Asan Hospital, University of Ulsan College of Medicine, Gangneung 25440, Republic of Korea

^6^Department of Chemical Engineering, Hongik University, Seoul 04066, Republic of Korea

^+^ These authors contributed equally to this work.

**Table of Contents**

**Supplementary Fig. 1**. Responses of the cohort to (**A**) tofacitinib and (**B**) baricitinib.

**Supplementary Fig. 2**. Availabilities of DAS 28 scores across the follow-up period.

**Supplementary Fig. 3**. Importance of the features used to predict positive response to (**A**) tofacitinib or (**B**) baricitinib.

**Supplementary Fig. 4.** Robustness of model discrimination across repeated data splits. (**A**) Distribution of ROC-AUC across 10 runs of stratified 5-fold cross-validation with shuffling (mean 0.791, SD 0.059) observed for the Tofacitinib model. (**B**) Analogous distribution (mean 0.798, SD 0.077) observed for the Baricitinib model.

**Supplementary Fig. 5.** SHAP (SHapley Additive exPlanations) analysis of ML model predictions.

**Supplementary Fig. 6.** Calibration of predicted probabilities for JAK inhibitor response. (**A**) Tofacitinib and (**B**) Baricitinib model.

**Supplementary Fig. 7.** Decision-curve analysis (DCA) comparing model-guided treatment selection with default strategies for (**A**) Tofacitinib and (**B**) Baricitinib model.

**Supplementary Fig. 8**. Multivariate analysis of the RA patients’ baseline characteristics with respect to a positive response to (**A**) tofacitinib and (**B)** baricitinib.

**Supplementary Table 1.** Baseline characteristics of the RA patients treated by tofacitinib in the training and test dataset.

**Supplementary Table 2.** Baseline characteristics of the RA patients treated by baricitinib in the training and test dataset.

**Supplementary Table 3**. Predictive performance of the tofacitinib model.

**Supplementary Table 4**. Predictive performance of the baricitinib model.

**
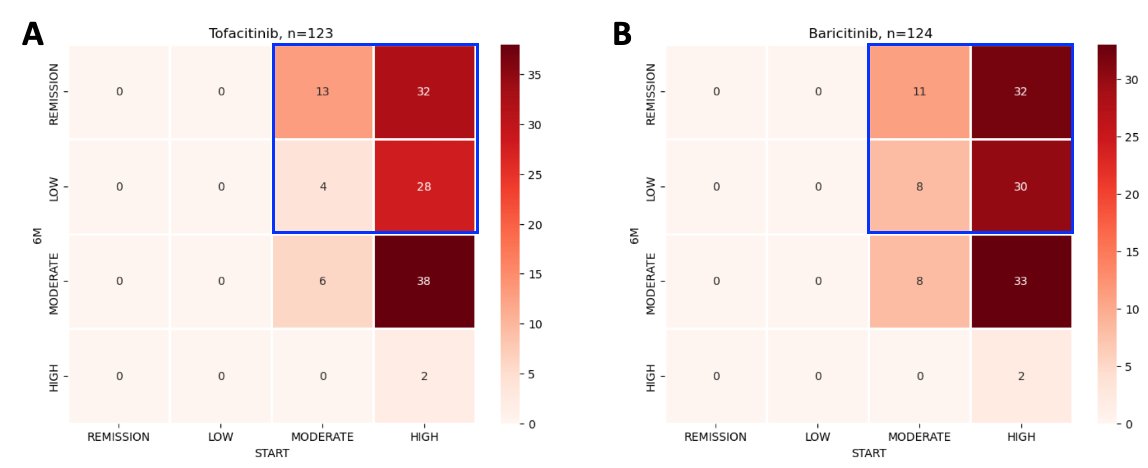
**

**Supplementary Fig. 1**. Responses of the cohort to (**A**) tofacitinib and (**B**) baricitinib. Response assessments were made after 6 months of the drug administration. Responses were assessed with respect to the changes in DAS28. The x-axis represents the status based on DAS28 at the index date (before administration of the drug) while the y-axis describes the status after 6 months of the drug administration. defined as the change in the status from moderate or high disease activity to low disease activity or remission (DAS28 <3.2). These correspond to the four tiles surrounded by the blue line in both figures. All other changes in the status were classified as lack of response including disease progression.

**
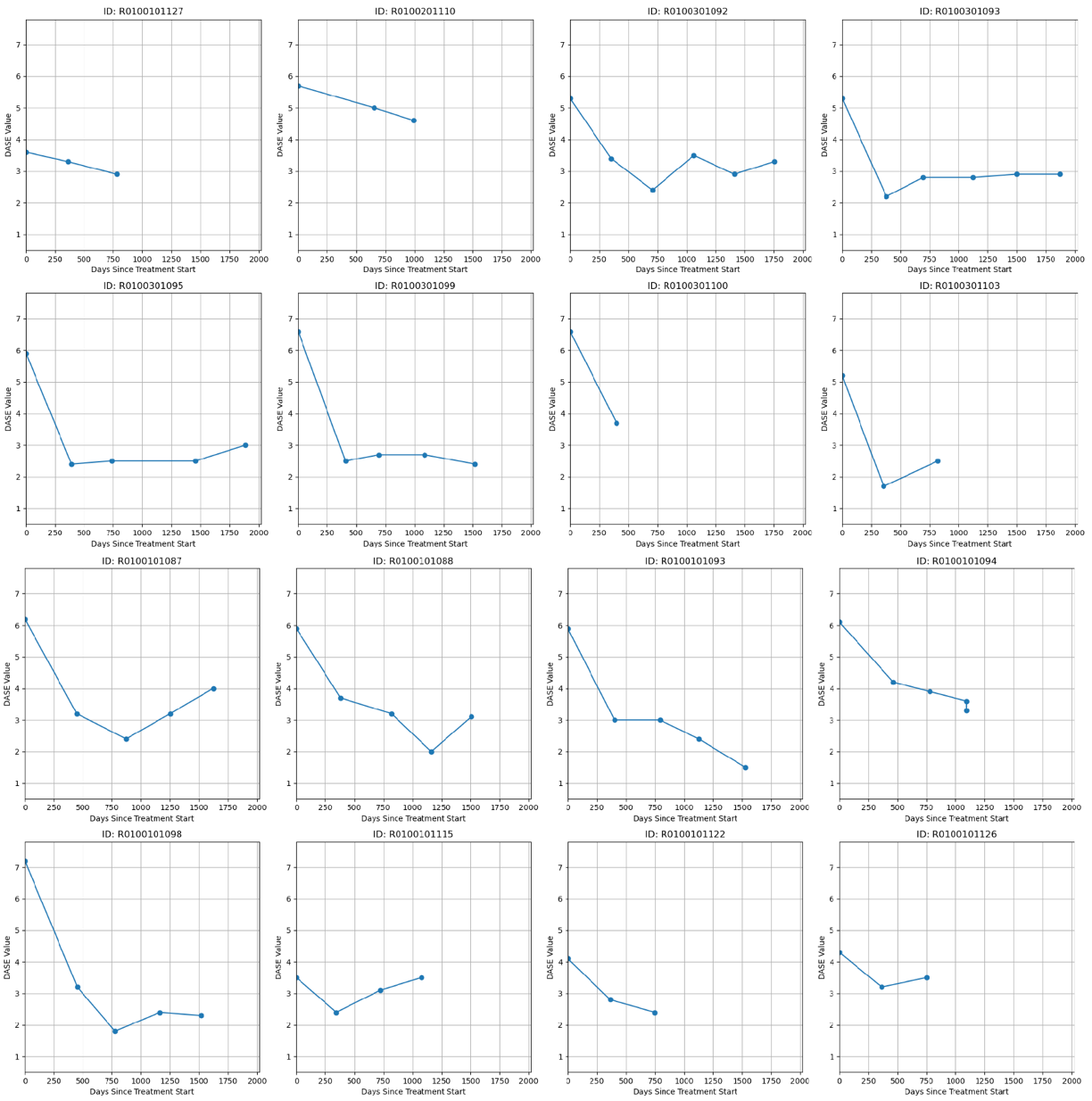
**

**Supplementary Fig 2.** Availabilities of DAS 28 scores across the follow-up period. Examples of dynamic changes in DAS 28 scores are presented for selected RA patients, illustrating the variability in both the timing and frequency of evaluation time points.

**
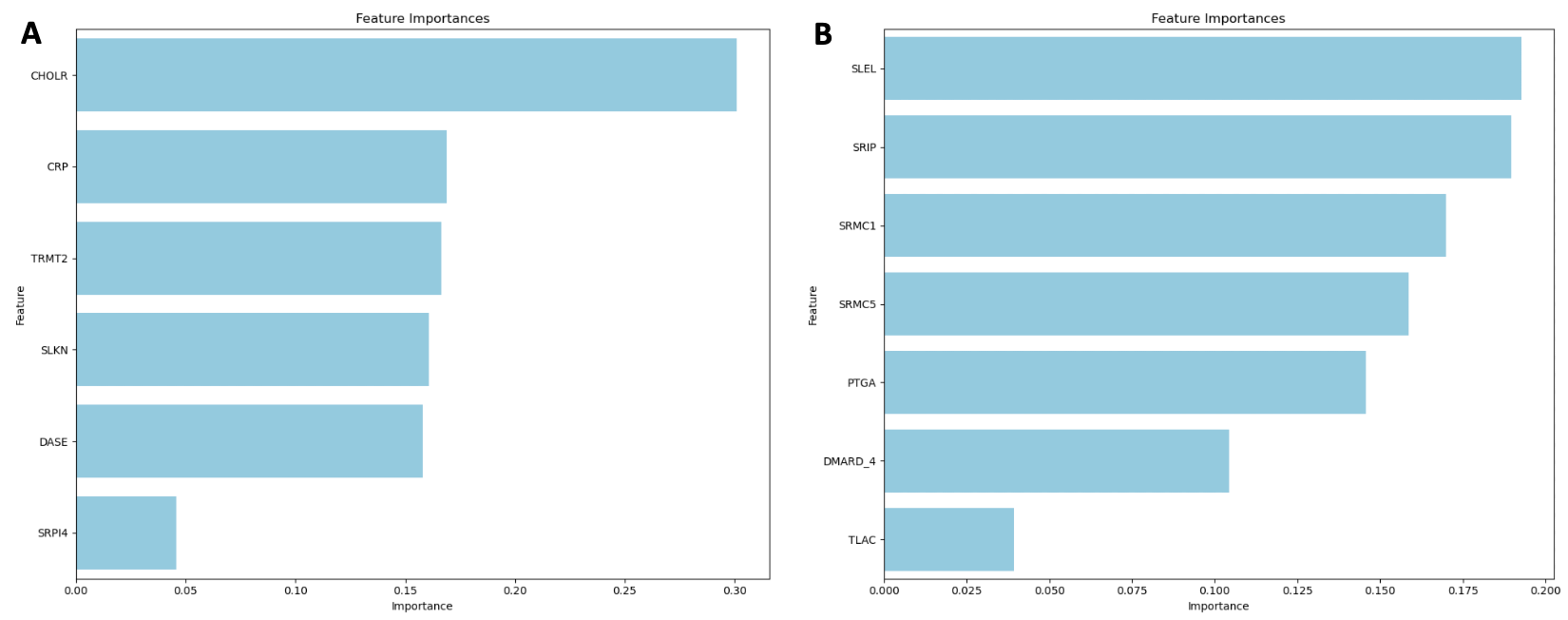
**

**Supplementary Fig. 3.** Importance of the features used to predict positive response to (**A**) tofacitinib or (**B**) baricitinib. The importance shown in x-axis have arbitrary unit with numeric values computed by the *PredictionValuesChange* method. Briefly, these values show how much on average the prediction changes if the feature value changes. The bigger the value of the importance, the bigger on average is the change to the prediction value, if this feature is changed. The importance values are normalized so that the sum of importances of all features is equal to 100. Abbreviations: CHOLR, cholesterol; CRP, C-reactive protein level; TRMT2, tenderness at the right 2^nd^ MTP joint; SLKN, swelling at the left knee joint; DASE, DAS 28-ERP at the index date; SRPI4, swelling at right 4^th^ PIP joint; SLEL, swelling at the left elbow joint; SRIP, swelling at the right IP joint; SRMC1, swelling at the right 1^st^ MCP joint; SRMC5, swelling at the right 5^th^ MCP joint; PTGA, patient’s global assessment of disease status; TLAC, tenderness at left AC joint.

**
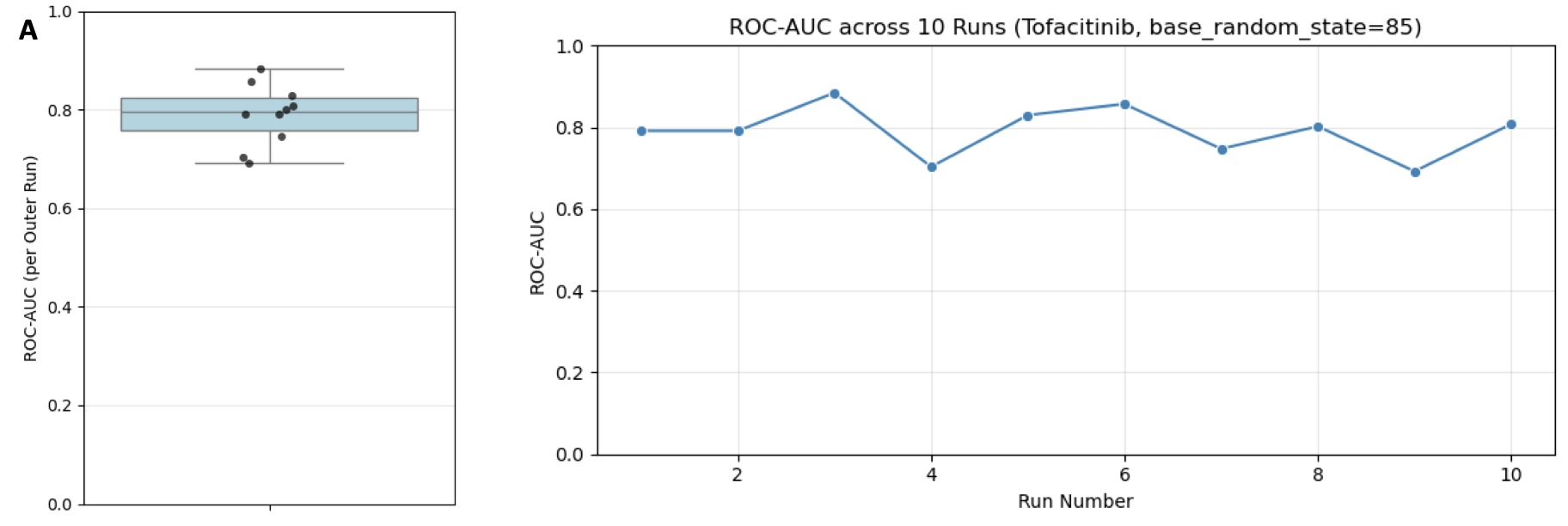
**

**
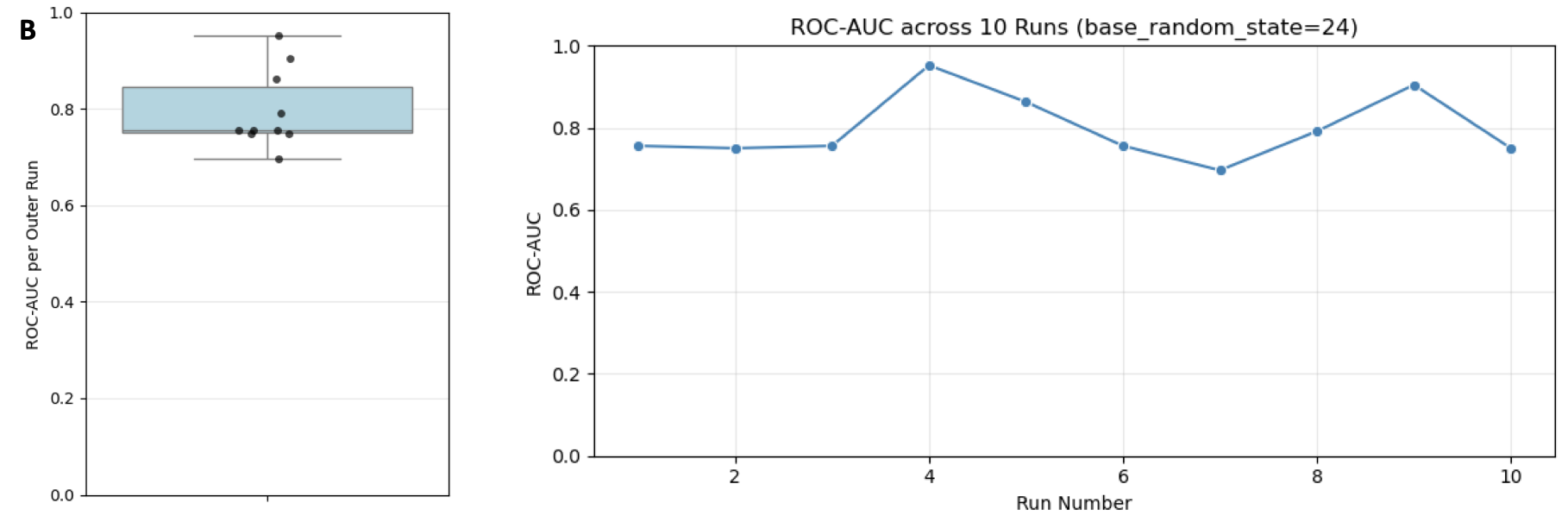
**

**Supplementary Fig. 4.** Robustness of model discrimination across repeated data splits. (**A**) Distribution of ROC-AUC across 10 runs of stratified 5-fold cross-validation with shuffling (mean 0.791, SD 0.059) observed for the Tofacitinib model. (**B**) Analogous distribution (mean 0.798, SD 0.077) observed for the Baricitinib model. Boxes show IQR with median; whiskers denote 1.5×IQR.

**
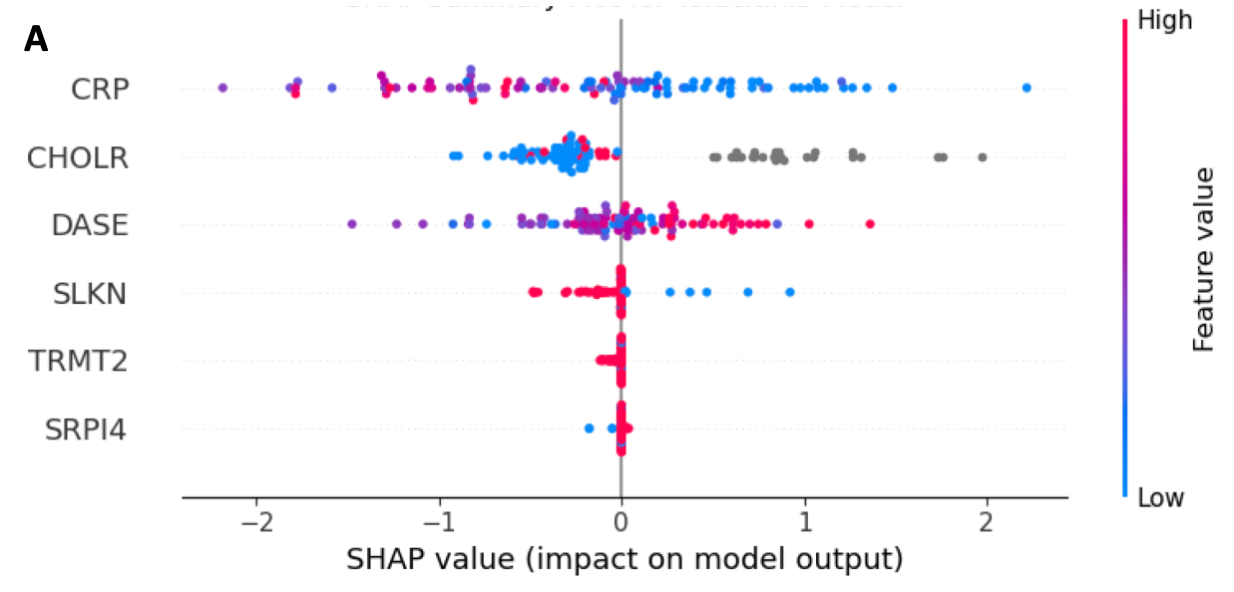

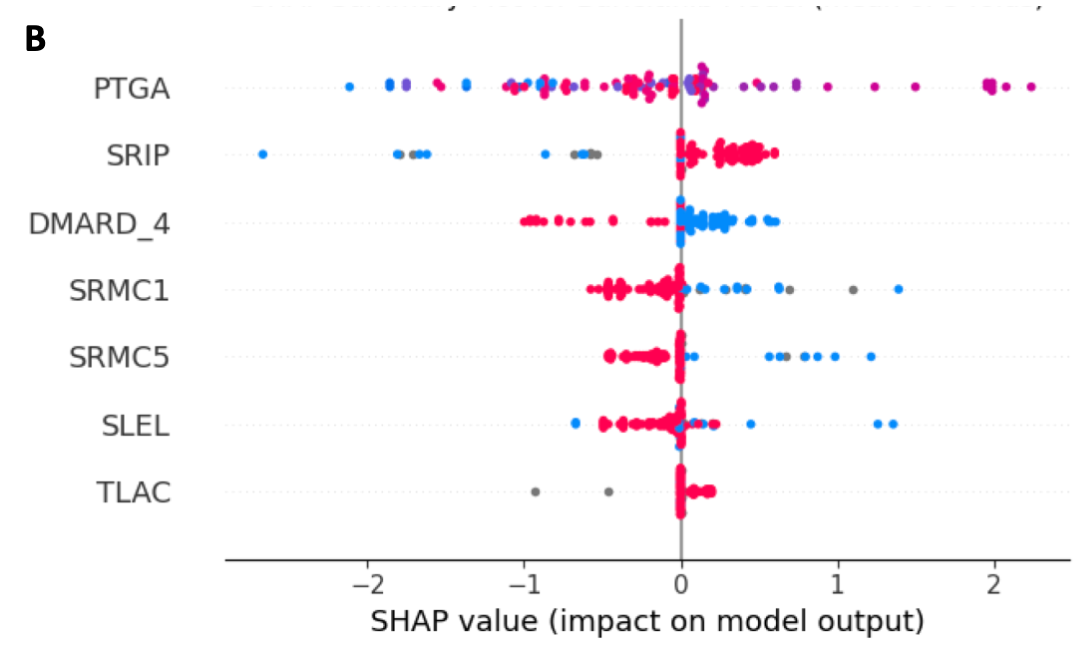
**

**
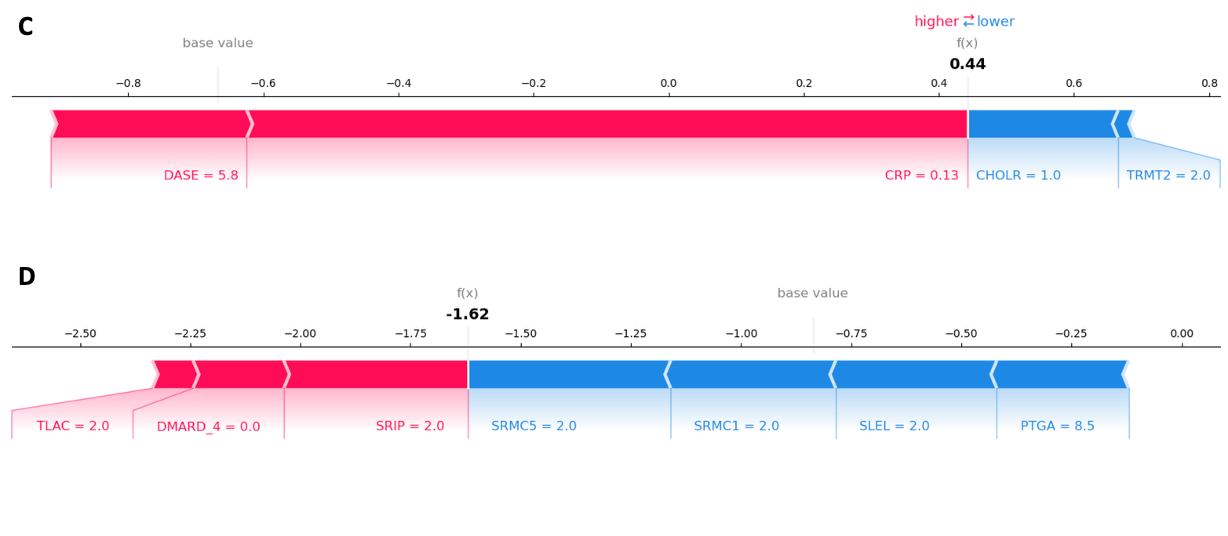
**

**Supplementary Figure 5.** SHAP (SHapley Additive exPlanations) analysis of ML model predictions. SHAP summary plots showing the overall contribution and direction of each covariate to predicted treatment response for the (**A**) tofacitinib and (**B**) baricitinib models. Each point represents an individual patient, with color indicating the magnitude of the covariate value (red = high, blue = low). Representative SHAP force plots illustrating patient-level interpretability of the ML models predicting response to (**C**) tofacitinib or (**D**) baricitinib. The plots demonstrate how individual features collectively contribute to the final prediction for selected patients, with red bars indicating features pushing the prediction toward a positive response and blue bars indicating features pushing it toward a negative response. Abbreviations: CHOLR, cholesterol; CRP, C-reactive protein level; TRMT2, tenderness at the right 2^nd^ MTP joint; SLKN, swelling at the left knee joint; DASE, DAS 28-ERP at the index date; SRPI4, swelling at right 4^th^ PIP joint; SLEL, swelling at the left elbow joint; SRIP, swelling at the right IP joint; SRMC1, swelling at the right 1^st^ MCP joint; SRMC5, swelling at the right 5^th^ MCP joint; PTGA, patient’s global assessment of disease status; TLAC, tenderness at left AC joint.

**
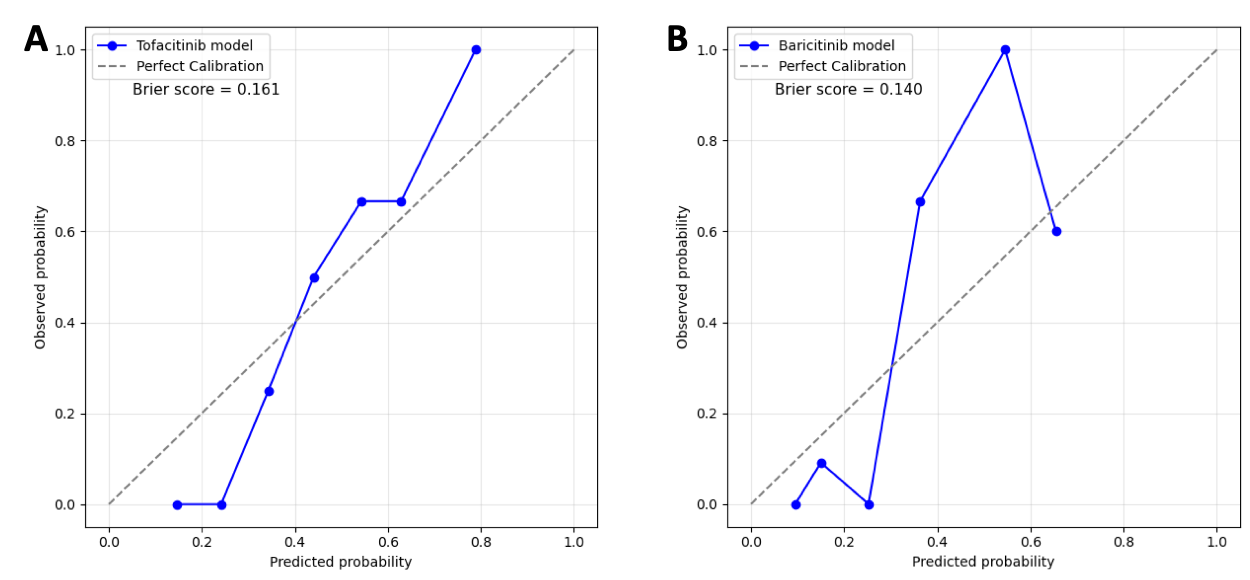
**

**Supplementary Figure 6.** Calibration of predicted probabilities for JAK inhibitor response. (**A**) Tofacitinib and (**B**) Baricitinib model. Reliability curves plot observed response frequency (y-axis) against mean predicted probability (x-axis) across 10 probability bins; the 45° line indicates perfect calibration. Curves were averaged over the stratified 5-fold cross-validation. Insets report Brier scores for each model.

**
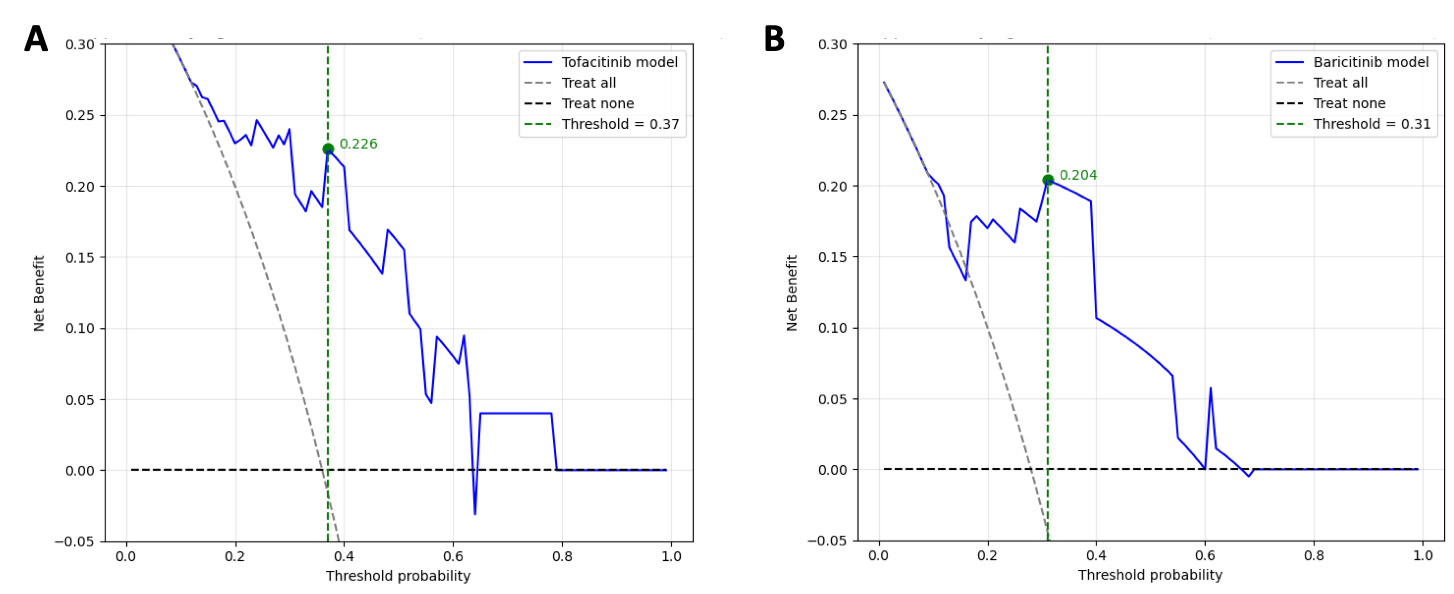
**

**Supplementary Figure 7.** Decision-curve analysis (DCA) comparing model-guided treatment selection with default strategies for (**A**) Tofacitinib and (**B**) Baricitinib model. Net benefit is plotted versus threshold probability, comparing the ML model (solid line) with “treat-all” and “treat-none” strategies (dashed lines). Curves aggregate results over the stratified 5-fold cross-validation. Net benefit was computed as $net benefit=\frac{TP}{N}-\frac{FP}{N}\frac{p_{t}}{1-p_{t}}$​​, $p_{t}$​ is the decision threshold; higher net benefit across clinically relevant thresholds indicates greater potential utility of probability-guided choices.

**
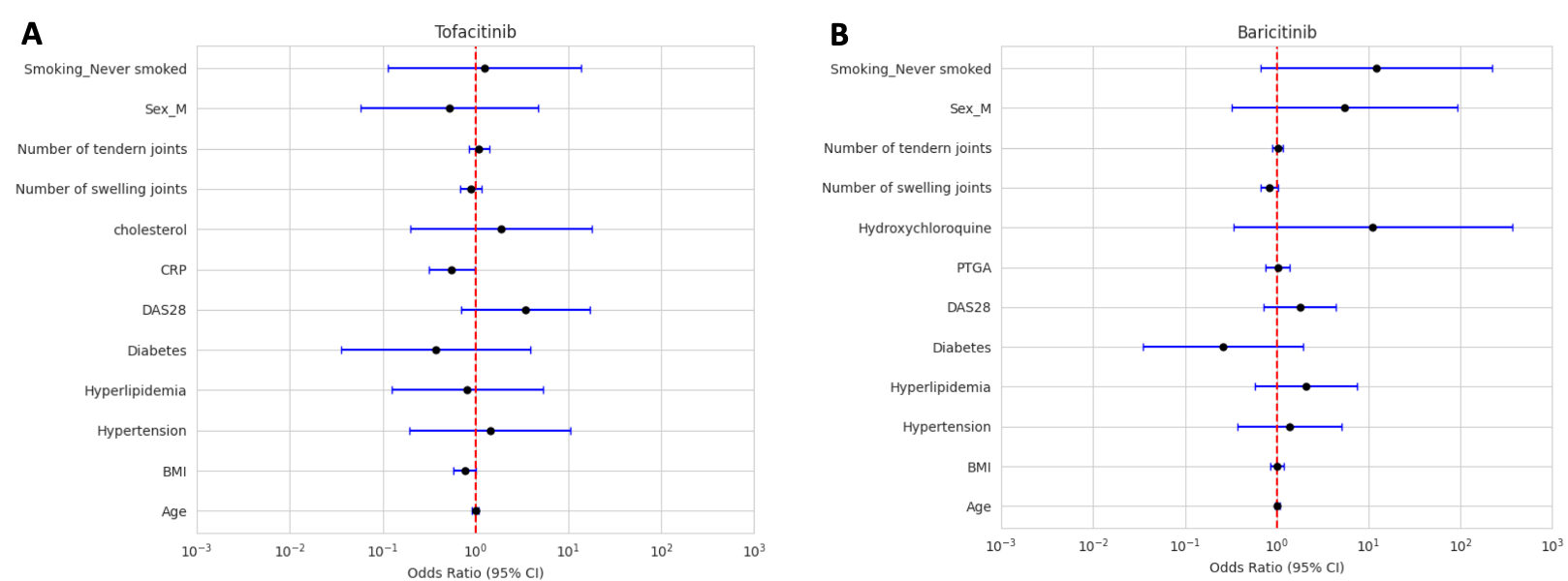
**

**Supplementary Fig. 8**. Multivariate analysis of the RA patients’ baseline characteristics with respect to a positive response to (**A**) tofacitinib and (**B)** baricitinib. The odds ratio (OR) values and 95% confidence intervals are presented based on the results of the multi-variate logistic regression.

**Supplementary Table 1.** Baseline characteristics of the RA patients treated by tofacitinib in the training and test dataset.

| **Characteristic** | **Training dataset**  (n = 98) | **Test dataset**  (n = 25) | ***P* value** |
| --- | --- | --- | --- |
| Age  ≤65 years  >65 years | 81 (83%)  17 (17%) | 21 (84%)  4 (16%) | 0.30 |
| Sex  Male  Female | 17 (17%)  81 (81%) | 7 (28%)  18 (72%) | 0.36 |
| BMI, kg/m^2^  <30  >30 | 92 (94%)  6 (6%) | 25 (100%)  0 (0%) | 0.13 |
| Smoking history  Ex-smoker  Current smoker  Never smoked | 6 (6%)  11 (11%)  81 (83%) | 2 (8%)  1 (4%)  22 (88%) | 0.54 |
| Comorbidities  Hypertension  Ischemic heart disease  Hyperlipidemia  Diabetes  Renal failure | 27 (28%)  2 (2%)  20 (20%)  12 (12%)  2 (2%) | 5 (20%)  0 (0%)  6 (24%)  4 (16%)  0 (0%) | 0.61  1.00  0.78  0.74  1.00 |
| csDMARDS at index date  any csDMARD  MTX only  csDMARDs excluding MTX | 92 (94%)  80 (82%)  50 (51%) | 23 (92%)  16 (64%)  16 (64%) | 0.12  0.06  0.25 |
| DAS 28 at index date |  |  | 0.20 |
| <2.6  2.6$\leq$DAS 28<3.2  3.2$\leq$DAS 28<5.1 | 0 (0%)  0 (0%)  21 (21%) | 0 (0%)  0 (0%)  2 (8%) |  |
| $\geq$5.1 | 77 (79%) | 23 (92%) |  |

**Supplementary Table 2.** Baseline characteristics of the RA patients treated by baricitinib in the training and test dataset.

| **Characteristic** | **Training dataset**  (n = 99) | **Test dataset**  (n = 25) | ***P* value** |
| --- | --- | --- | --- |
| Age  ≤65 years  >65 years | 81 (82%)  18 (18%) | 17 (68%)  8 (32%) | 0.90 |
| Sex  Male  Female | 16 (16%)  83 (84%) | 4 (16%)  21 (84%) | 1.00 |
| BMI, kg/m^2^  <30  >30 | 96 (97%)  3 (3%) | 23 (92%)  2 (8%) | 0.72 |
| Smoking history  Ex-smoker  Current smoker  Never smoked | 10 (10%)  10 (10%)  79 (80%) | 1 (4%)  2 (8%)  22 (88%) | 0.58 |
| Comorbidities  Hypertension  Ischemic heart disease  Hyperlipidemia  Diabetes  Renal failure | 30 (30%)  4 (4%)  28 (28%)  9 (10%)  0 (0%) | 2 (8%)  0 (0%)  6 (24%)  1 (4%)  0 (0%) | 0.02  0.58  0.80  0.68 |
| csDMARDS at index date  any csDMARD  MTX only  csDMARDs excluding MTX | 88 (89%)  77 (78%)  49 (49%) | 25 (100%)  22 (88%)  13 (52%) | 0.08  0.26  0.82 |
| DAS 28 at index date |  |  | 0.15 |
| <2.6  2.6$\leq$DAS 28<3.2  3.2$\leq$DAS 28<5.1 | 0 (0%)  0 (0%)  19 (19%) | 0 (0%)  0 (0%)  8 (32%) |  |
| $\geq$5.1 | 80 (81%) | 17 (68%) |  |

**Supplementary Table 3**. Predictive performance of the tofacitinib model.

| **Performance metric** | **Validation** (n=98) | **Test** (n=25) |
| --- | --- | --- |
| Accuracy | 0.796 | 0.800 |
| Sensitivity | 0.765 | 0.889 |
| Specificity | 0.813 | 0.750 |
| Positive Predictive Value | 0.684 | 0.667 |
| Negative Predictive Value | 0.867 | 0.923 |

**Supplementary Table 4**. Predictive performance of the baricitinib model.

| **Performance metric** | **Validation** (n=99) | **Test** (n=25) |
| --- | --- | --- |
| Accuracy | 0.818 | 0.880 |
| Sensitivity | 0.800 | 0.857 |
| Specificity | 0.826 | 0.889 |
| Positive Predictive Value | 0.667 | 0.750 |
| Negative Predictive Value | 0.905 | 0.941 |
